# Generation and Starch Characterization of Non-Transgenic BEI and BEIIb Double Mutant Rice (*Oryza sativa*) with Ultra-High Level of Resistant Starch

**DOI:** 10.1186/s12284-020-00441-0

**Published:** 2021-01-06

**Authors:** Satoko Miura, Nana Koyama, Naoko Crofts, Yuko Hosaka, Misato Abe, Naoko Fujita

**Affiliations:** Department of Biological Production, Akita Prefectural University, Akita, 010-0195 Japan

**Keywords:** Resistant starch, Rice (*Oryza sativa*), Starch branching enzyme, Endosperm starch, Amylopectin, Amylose

## Abstract

**Background:**

Cereals high in resistant starch (RS) are gaining popularity, as their intake is thought to help manage diabetes and prediabetes. Number of patients suffering from diabetes is also increasing in Asian countries where people consume rice as a staple food, hence generation of practically growable high RS rice line has been anticipated. It is known that suppression of starch branching enzyme (BE) IIb increases RS content in cereals. To further increase RS content and for more practical use, we generated a non-transgenic *be1 be2b* double mutant rice (*Oryza sativa*) line, which completely lacked both proteins, by crossing a *be1* mutant with a *be2b* mutant.

**Results:**

The *be1 be2b* mutant showed a decrease in intermediate amylopectin chains and an increase in long amylopectin chains compared with *be2b*. The amylose content of *be1 be2b* mutant (51.7%) was the highest among all pre-existing non-transgenic rice lines. To understand the effects of chewing cooked rice and cooking rice flour on RS content, RS content of mashed and un-mashed cooked rice as well as raw and gelatinized rice flour were measured using *be1 be2b* and its parent mutant lines. The RS contents of mashed cooked rice and raw rice flour of *be1 be2b* mutant (28.4% and 35.1%, respectively) were 3-fold higher than those of *be2b* mutant. Gel-filtration analyses of starch treated with digestive enzymes showed that the RS in *be1 be2b* mutant was composed of the degradation products of amylose and long amylopectin chains. Seed weight of *be1 be2b* mutant was approximately 60% of the wild type and rather heavier than that of *be2b* mutant.

**Conclusions:**

The endosperm starch in *be1 be2b* double mutant rice were enriched with long amylopectin chains. This led to a great increase in RS content in cooked rice grains and rice flour in *be1 be2b* compared with *be2b* single mutant. *be1 be2b* generated in this study must serve as a good material for an ultra-high RS rice cultivar.

**Supplementary Information:**

The online version contains supplementary material available at 10.1186/s12284-020-00441-0.

## Background

Starch is produced from photosynthetic products through biological reactions and serves as an important carbohydrate source for heterotrophs. Starch is utilized not only in food industries but also in technological and medical industries, and the applications of starch are expected to expand further in future. Starch is of two types: one type is easily degraded by digestive enzymes, while the other type, known as resistant starch (RS), is less susceptible to digestive enzymes, travels to the colon as a high molecular weight of partially digested starch, and functions similar to fiber (Englyst et al. 1992; Nugent 2005; Matsuki 2010). RS is further categorized into five classes (RS1–5), depending on its characteristics: RS1 is physically inaccessible to digestive enzymes because of thick cell walls; RS2 shows B-type crystal structure, based on the X-ray diffraction pattern; RS3 is retrograded starch, which is initially gelatinized and then recrystallized to form a stable structure; RS4 is chemically modified by esterification or etherification to reduce digestibility; and RS5 are starch granules that have been modified with lipids forming a starch -lipid complex (Englyst et al. 1992; Brown et al. 1995; Hasjim et al. 2010; Matsuki 2010). Intake of foods high in RS content suppresses an acute increase in blood sugar level and insulin response, and prolongs fullness (Nugent 2005; Matsuki 2010). Suppression of insulin response likely promotes fat burn, thus preventing lifestyle-related diseases such as diabetes (Wilcox 2005).

Amylopectin is a branched glucose polymer that represents the major component of starch. Branches of amylopectin are generated by starch branching enzymes (BEs) (Nishi et al. 2001; Nakamura 2002; Satoh et al. 2003b). Three BE isozymes exist in rice (*Oryza sativa*) and maize (*Zea mays*): BEI, BEIIa, and BEIIb. Among these isozymes, BEIIb play a major role in amylopectin branching and is strongly expressed in endosperm and kernels (Boyer and Preiss 1978; Dang and Boyer 1988; Ohdan et al. 2005). Single mutants of *BEI*, *BEIIa*, and *BEIIb* gene have been isolated in rice (Nishi et al. 2001; Nakamura 2002; Satoh et al. 2003a; Satoh et al. 2003b) and maize (Boyer and Preiss 1978; Blauth et al. 2001; Blauth et al. 2002). Rice *be2b* single mutant plants produce opaque seeds that weigh less than wild-type (WT) seeds (Nishi et al. 2001; Matsushima et al. 2012). Amylopectin chain length distribution analyses show that the *be2b* single mutants produce significantly less short amylopectin chains, with degree of polymerization (DP) = 6–12 (Nishi et al. 2001; Asai et al. 2014; Nakata et al. 2018); therefore, BEIIb is thought to produce branch points of A and B_1_ chains of amylopectin. The reduction of starch synthase I (SSI) activity is accompanied by the loss of BEIIb in rice (Nishi et al. 2001). The *be2b* mutant exhibits a reduction in amylopectin synthesis and consequently an increase in the proportion of amylose compared with the WT (Asai et al. 2014). In addition, the gelatinization temperature of starch is higher in the *be2b* mutant because of the increase in intermediate amylopectin chains with DP = 14-24 (Tanaka et al. 2004).

By contrast, the rice *be2a* mutant shows no significant difference in seed morphology and amylopectin chain length distribution compared with the WT (Nakamura 2002). Analysis of the recombinant BEIIa enzyme suggests that the function of BEIIa partially overlaps with or is complemented by BEI and/or BEIIb (Nakamura 2002). On the other hand, rice *be1* mutant shows a slight decrease in long amylopectin chains (DP > 37) and intermediate chains (DP = 12–21), and a slight increase in short amylopectin chains (DP < 10), suggesting that BEI is involved in the generation of branch points of B_1_, B_2_, and B_3_ chains (Satoh et al. 2003b). However, loss of BEI causes a very small change in amylose content, although the gelatinization temperature of *be1* is lower than that of the WT (Satoh et al. 2003b; Abe et al. 2013; Abe et al. 2014).

Introduction of the rice *BEIIb* gene into the *be2b* null mutant of rice produced a variety of lines with different *BEIIb* expression levels and diverse endosperm starch properties (Tanaka et al. 2004). Low expression of *BEIIb* results in less amylopectin short chains (DP < 16) (Tanaka et al. 2004). On the other hand, overexpression of *BEIIb* leads to the accumulation of excessively branched, water-soluble glucans enriched in amylopectin short chains (DP < 16) (Tanaka et al. 2004). Taken together, these data show that the loss of BEIIb greatly affects starch properties, and the function of BEIIb cannot be compensated for by other BEs (BEI and BEIIa), indicating that BEIIb is indispensable. Furthermore, the loss of BEIIb drastically elevates RS content to much higher orders of magnitude than the RS content of high amylose rice cultivars such as *indica* rice and the *ss3a* mutant (Zhou et al. 2016). This indicates that the increase in amylopectin long chains leads to a greater increase in the RS content than that caused by the increase in amylose content (Tsuiki et al. 2016).

The maize *be2b* mutant (amylomaize) has higher amylose content than the rice *be2b* mutant (Boyer et al. 1976; Nishi et al. 2001). Remarkably, the maize GEMS-0067 lines, which was developed using germplasm obtained from USDA-ARS Germplasm Enhancement of Maize (GEM) project (Li et al. 2008), show extraordinarily high values of many parameters: apparent amylose content = 83.1–85.6%; final gelatinization temperature = 122.0–130.0 °C; RS content = 30.9–34.3%. The maize GEMS-0067 lines are thought to be *be1 be2b* double mutants (Personal communication with Prof. Jane) (Li et al. 2008). The expression of *BEI* and *BEIIb* has been down-regulated in rice (Zhu et al. 2012; Wang et al. 2017; Pan et al. 2018; Sawada et al. 2018), barley (*Hordeum vulgare*) (Carciofi et al. 2012), and wheat (*Triticum aestivum*) (Regina et al. 2006). In *indica* rice, down-regulation of *BEI* and *BEIIb* genes resulted in higher apparent contents of amylose (64.8%) and RS (14.6%) than the WT (Zhu et al. 2012). Simultaneous knockdown of multiple *BE* genes likely suppressed amylopectin biosynthesis, which in turn enhanced amylose synthesis. In addition, the ratio of long amylopectin branches was also higher. Amylose was preferentially degraded in germinating seeds compared with the long amylopectin chains (Zhu et al. 2012; Wang et al. 2017; Pan et al. 2018). In *japonica* rice, down-regulation of both *BEI* and *BEIIb* genes resulted in much lower seed weight, with fewer intermediate chains (DP = 11–22), compared with the down-regulation of *BEIIb* alone (Sawada et al. 2018). In transgenic barley, suppression of all three *BEs* resulted in amylose as the only endosperm starch and a very high RS content of gelatinized starch (65%) (Carciofi et al. 2012). In transgenic wheat, suppression of *BEIIb* alone did not affect the amylose content; however, suppression of both *BEIIa* and *BEIIb* genes resulted in high amylose content (> 70%) (Regina et al. 2006). These studies suggest that suppression of multiple BE isozymes, rather than BEIIb alone, results in less amylopectin short chains and consequently higher amylose and RS contents.

Increasing the RS content of cereals is receiving considerable attention; however, factors that affect the digestion of starch upon intake, such as whether the cereals were cooked/raw and mashed/un-mashed, need to be studied further. The structure and composition of native starch, such as amylose content and branch length of amylopectin, have been analyzed in high RS cereals; however, the structure of residual glucans after treatment with digestive enzymes remains largely unknown. To determine the type of glucan structure that is beneficial for gastrointestinal health, it is important to understand the structure of RS and residual glucan.

In this study, we generated a non-transgenic *be1 be2b* double mutant rice line, which completely lacked both BEI and BEIIb proteins. The structure of endosperm starch in the double mutant was analyzed to examine its relationship with the RS content of raw/gelatinized rice flour and mashed/un-mashed cooked rice grains. Furthermore, structures of RS in raw rice flour and mashed cooked rice prepared from high RS rice lines, *be2b* and *be1 be2b*, were analyzed, and the RS structure was discussed.

## Results

### Absence of BEI and BEIIb

**Fig. 1:**
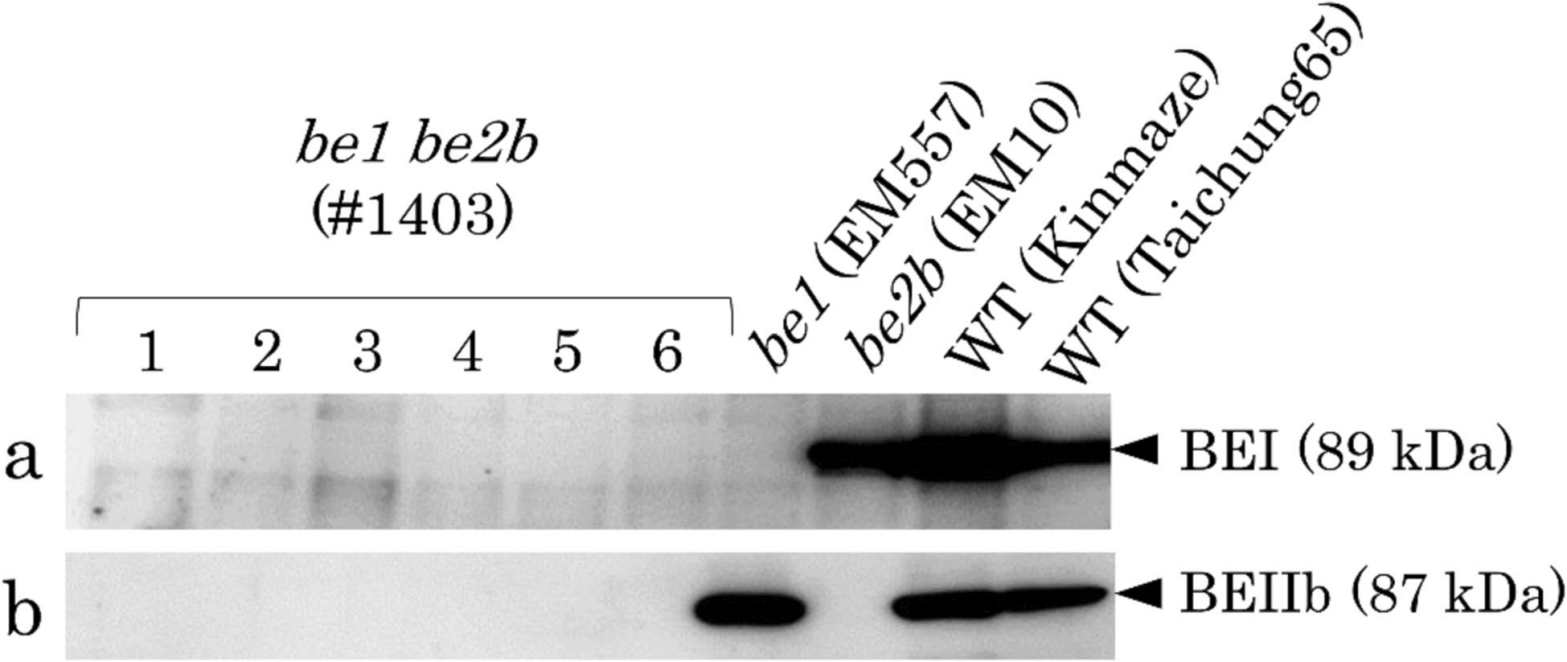
Western blotting analysis of starch branching enzymes, BEI and BEIIb. Total proteins extracted from mature rice endosperm of *be1* *be2b* double mutant, its parental mutants, and WT were analyzed by western blotting. (**a**, **b**) Western blotting analyses using anti-BEI antibody (**a**) and anti-BEIIb antibody (**b**). Proteins extracts were prepared from six endosperms of one *be1* *be2b* plant

Total proteins were extracted from the *be1 be2b* double mutant (#1403), its parental single mutants *be1* (EM557) and *be2b* (EM10), and WT cultivars (Taichung 65 and Kinmaze), and BEI and BEIIb proteins were detected by western blotting (Fig. 1). When BEI antibody was used, BEI signal was detected in WT cultivars and *be2b* single mutant, but not in *be1* single mutant and *be1 be2b* double mutant (Fig. 1). Similarly, when BEIIb antibody was used, BEIIb signal was detected in WT cultivars and *be1* single mutant but not in *be2b* and *be1 be2b* (Fig. 1). This clearly showed that the mutation sites in *be1* and *be2b* (see Plant materials) lead to the deficiencies of corresponding proteins.

### Seed Morphology and Seed Weight

**Fig. 2:**
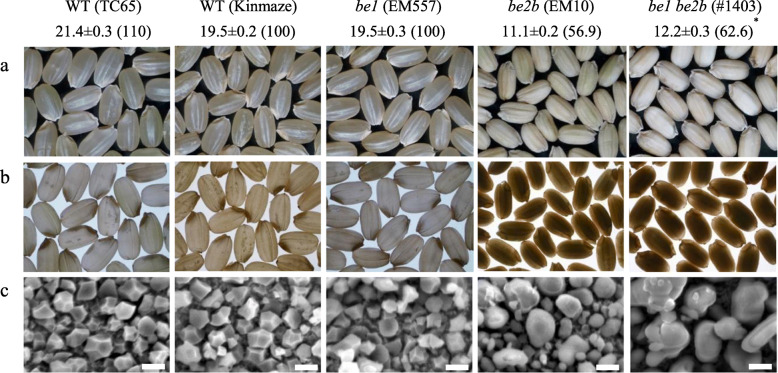
Seed morphology, seed weight, and starch granule morphology. Photos of dehulled mature seeds of the wild type (WT), *be1* and *be2b* single mutants, and *be1* *be2b* double mutant were captured under light from above (**a**) and beneath (**b**). Numbers above the photos indicate mg of seed weight. Data represent mean ± standard error (SE; *n* = 50). Values in parentheses indicate the percentage of seed weight relative to the WT rice cultivar Kinmaze. Significant difference between *be2b* and *be1* *be2b* are indicated with an asterisk (*P < 0.05; Student’s t-test). (**c**) Morphology of a purified starch granule observed by scanning electron microscopy. Scale bars: 5 μm

Seeds of WT cultivars and *be1* single mutant were translucent, while those of the *be2b* single mutant and *be1 be2b* double mutant were opaque (Fig. 2a, b). The weight of *be1* seeds was 19.5 mg, which was similar to that of WT seeds (Taichung 65, 21.4 mg). On the other hand, the seed weight of *be2b* was 56.9% (11.1 mg) of the WT (Kinmaze), and that of the *be1 be2b* double mutant (12.2 mg) was 62.6% and 57.0% of Kinmaze and Taichung 65, respectively, which were regarded as WT. The seed weight of *be1 be2b* was significantly heavier than that of *be2b* (Fig. 2).

**Fig. 3:**
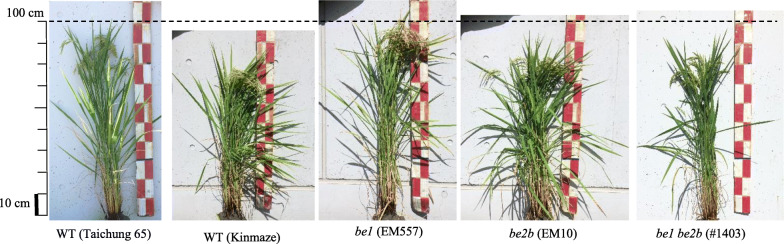
Photos of typical rice plants at developing stage. Bars indicate 10 cm

Fertility rate of the double mutant, its parental mutants and the WTs for the past 3 years were calculated (Table 1). There were no significant differences among the lines in 2017. However, the fertility rate of the *be1* single mutant were statistically lower than the WT Kinmaze in 2018 and Taichung 65 in 2019. While there were no significant differences in fertility rate between *be1 be2b* and other lines. In addition, growth of the plants was similar (Fig. 3), and there were no significant differences in number of panicles among the analyzed lines (Table 1). Length of culm tended to be dependent on the cultivar; Kinmaze and *be2b* were short, while Taichung 65 and *be1* were long. Culm length of *be1 be2b* was similar to that of Kinmaze and *be2b*. Therefore, the growth of *be1 be2b* was not impeded by the absence of two BE enzymes.

**Table 1:** Fertility rate for 3 years and panicle numbers and culm length of plants

Genotype	Fertility rate (%)^a^			Panicle number^b^	Culm length^b^
	‘2017	‘2018	‘2019		
WT (Taichung 65)	93.5 ± 1.5c	88.5 ± 0.4 cd	93.0 ± 1.3c	17.4 ± 1.0cd	81.2 ± 1.0c
WT (Kinmaze)	96.0 ± 0.2c	90.5 ± 1.3c	87.8 ± 0.7 cd	18.6 ± 1.1cd	57.8 ± 0.9e
*be1* (EM557)	85.1 ± 2.3c	71.7 ± 0.9d	79.0 ± 0.6d	16.2 ± 1.2d	82.5 ± 1.4c
*be2b* (EM10)	89.0 ± 2.4c	80.0 ± 4.9 cd	87.9 ± 1.6 cd	22.0 ± 0.9c	67.8 ± 1.2d
*be1 e2b* (#1403)	96.1 ± 0.3c	90.1 ± 2.0 cd	86.1 ± 2.6 cd	19.9 ± 0.7cd	71.4 ± 1.7d

Data represent mean ± standard error (*n* = 3^a^, *n* = 10^b^)

Different lowercase letters (c-e) indicate significant differences among rice genotypes (*P* < 0.05; Tukey-Kramer method)

### Morphology, Crystallinity, and Thermal Properties of Starch Granules

Starch granule morphology, crystallinity, and thermal properties were analyzed using purified starch granules. The starch granules of WT cultivars and *be1* single mutant observed by SEM were polygonal in shape and 2–5 μm in size (Fig. 2c). By contrast, *be2b* and *be1 be2b* mutants showed larger irregularly shaped starch granules that formed aggregates within the amyloplast (Fig. 2c; Asai et al. 2014). Peanut-like rod-shaped starch granules were also observed in *be1 be2b*. High amylose content lines, such as rice *ss3a be2b* and maize *be1 be2b* double mutants, also possess irregular rod-shaped starch granules, indicating that irregularly shaped starch granules may be one of the characteristic features of lines with high amylose content (Jiang et al. 2010; Wei et al. 2010; Zhu et al. 2012; Asai et al. 2014).

**Fig. 4:**
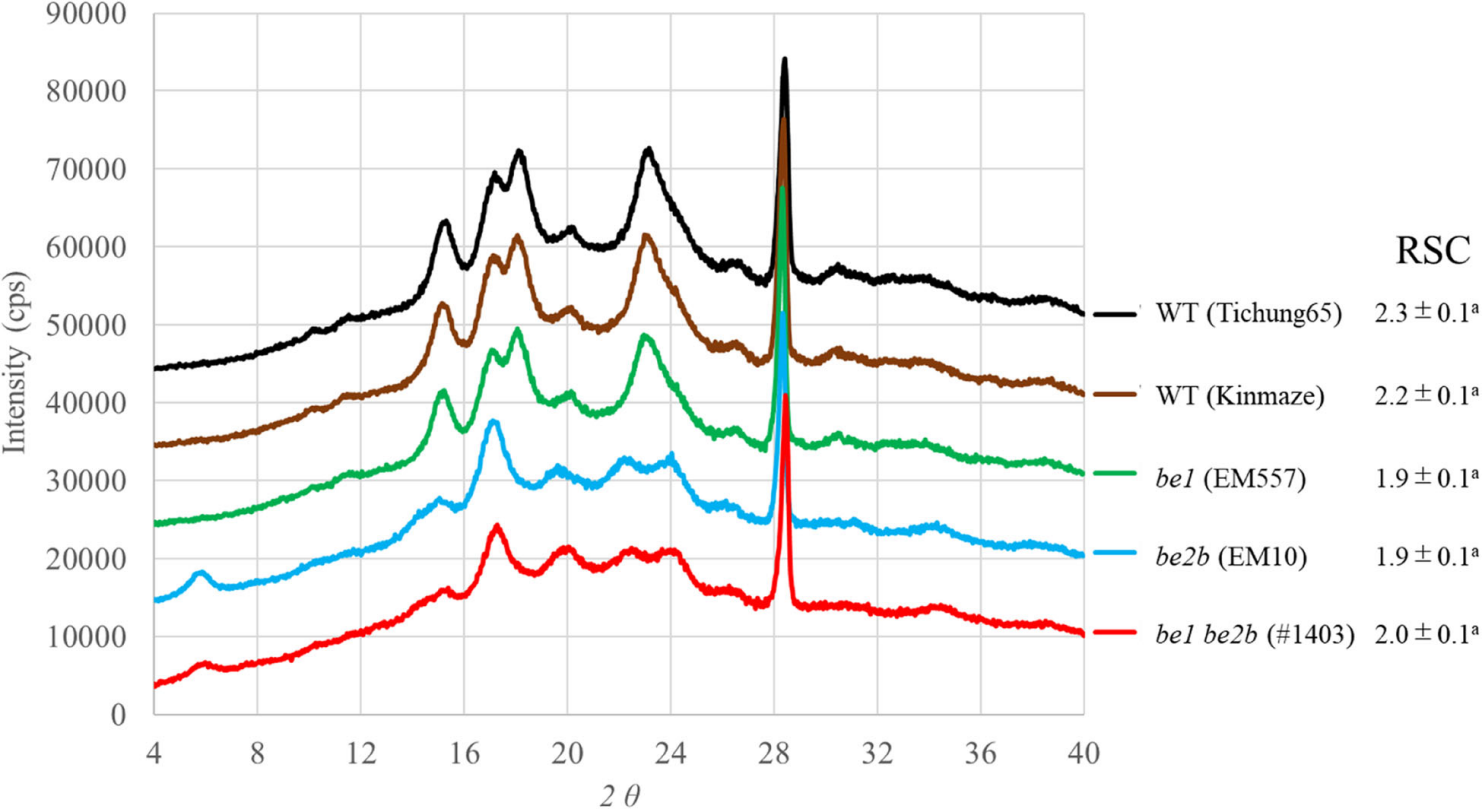
Typical X-ray diffraction patterns and their relative starch crystallinity (RSC) of starch. Starch isolated from endosperm in WT cultivars, *be1*, *be2b*, and *be1* *be2b* were analyzed. There is no significant difference in RSC among rice lines by Tukey-Kramer method (*P* < 0.05)

Analysis of crystallinity of purified starch granules by X-ray diffraction measurements revealed that starch granules of WT cultivars and *be1* single mutant showed a typical A-type crystal structure, while those of *be2b* and *be1 be2b* showed a typical B-type crystal structure (Fig. 4). To calculate the relative starch crystallinity (RSC), calcium fluoride (CaF_2_) was added as an internal standard (2θ = 28.3°). There were no significant differences in RSC among rice lines by Tukey-Kramer method (*P* < 0.05) (Fig. 4).

The thermal properties of starch were analyzed by DSC (Table 2). The onset, peak, and conclusion gelatinization temperatures of *be1* were 5.4 °C, 4.2 °C, and 3.2 °C lower, respectively, than those of the parental WT cultivar Taichung 65; by contrast, the corresponding values of *be2b* were 6.8 °C, 16.3 °C, and 23.8 °C higher, respectively, than those of the parental WT cultivar Kinmaze. These values are consistent with previous studies (Satoh et al. 2003b; Tanaka et al. 2004). Strikingly, the onset, peak, and conclusion gelatinization temperatures of *be1 be2b* were 9.2 °C, 7.5 °C, and 2.2 °C higher, respectively, than those of *be2b*. The required thermal energy of *be1* (10.0 mJ mg^− 1^) was slightly less than that of Taichung 65 (11.3 mJ mg^− 1^), whereas that of *be2b* (17.6 mJ mg^− 1^) was considerably greater than that of Kinmaze (9.6 mJ mg^− 1^). By contrast, the required thermal energy of *be1 be2b* (7.1 mJ mg^− 1^) was much less than that of *be2b* (Table 2), possibly because the amylose content of *be1 be2b* was higher than that of *be2b* (described below).

**Table 2:** Thermal properties of endosperm starch in different rice genotypes determined by differential scanning calorimetry (DSC)

Genotype	Thermal properties			
	T_*o*_ (°C)	T_*p*_ (°C)	T_*c*_ (°C)	ΔH (mJ mg^−1^)
WT (Taichung 65)	51.0 ± 0.1b	58.0 ± 0.0c	64.1 ± 0.1b	11.3 ± 0.1ab
WT (Kinmaze)	45.4 ± 0.0c	53.0 ± 0.1d	59.7 ± 0.0b	9.6 ± 0.2b
*be1* (EM557)	45.6 ± 0.3c	53.8 ± 0.1d	60.9 ± 0.1b	10.0 ± 0.4b
*be2b* (EM10)	52.2 ± 0.6b	69.3 ± 0.4b	83.5 ± 1.6a	17.6 ± 2.0a
*be1 be2b* (#1403)	61.4 ± 1.3a	76.8 ± 0.2a	85.7 ± 0.7a	7.1 ± 0.6b

Data represent mean ± standard error (*n* = 3). Different lowercase letters (a-d) indicate significant differences among rice genotypes (*P* < 0.05; Tukey-Kramer method). T*o*, onset temperature; T*p*, peak temperature; T*c*, conclusion temperature; ΔH, gelatinization enthalpy of starch

### Analyses of Starch Structure

**Fig. 5:**
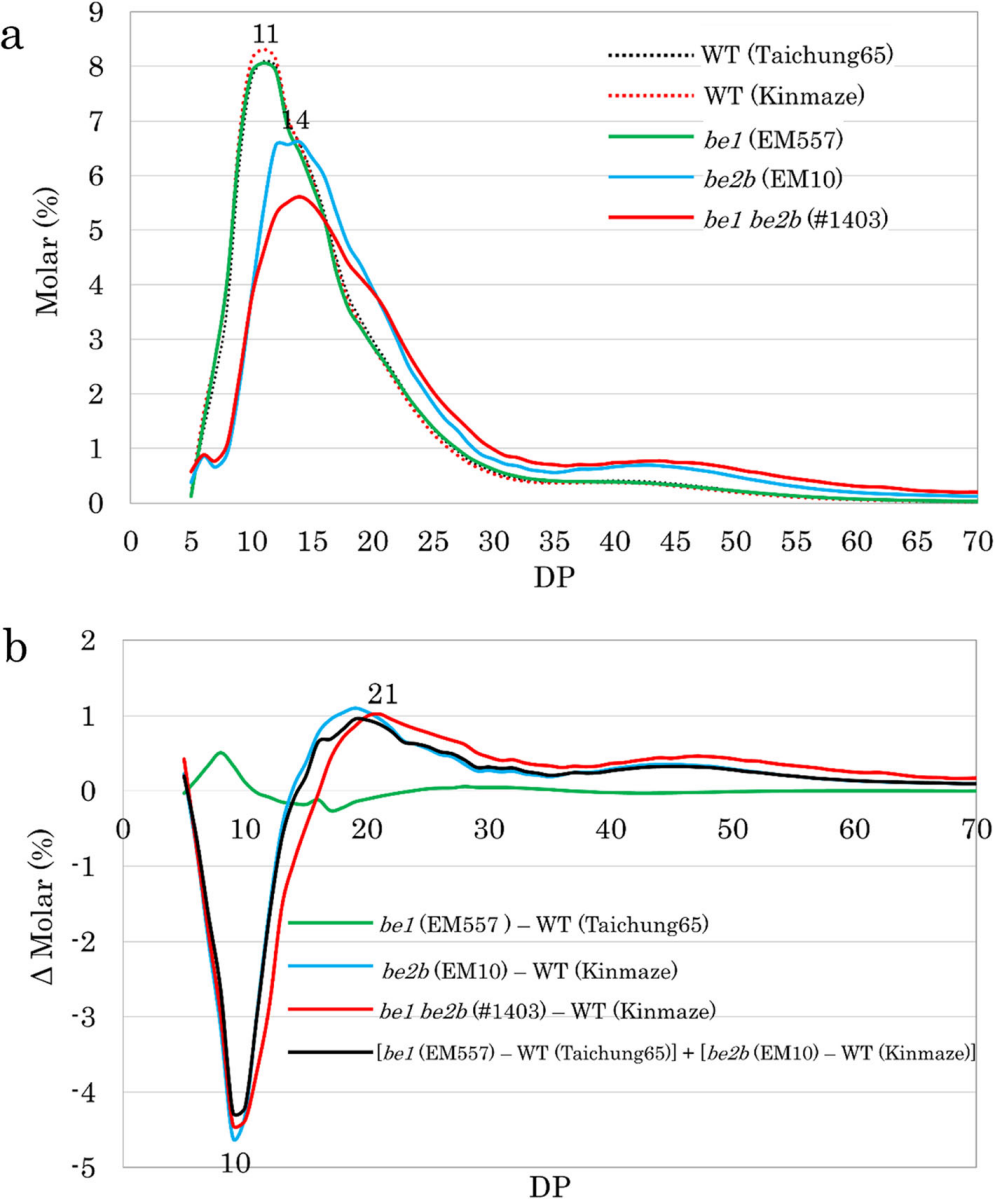
Chain length distribution of endosperm amylopectin analyzed by capillary electrophoresis. **a** Chain length distribution patterns of WT, *be1*, *be2b*, and *be1* *be2b*. **b** Differences in the fine structure of amylopectin between the WT and mutant lines. Differences are shown as Δ Molar %, and the value was calculated by subtracting the pattern of WT from each mutant line, as indicated. Black line indicates a theoretical value calculated by adding the effects of the loss of BEI alone and BEIIb alone

To determine the basis of differences observed in the crystallinity and gelatinization temperatures between *be1 be2b*, parental mutant lines, and the WT, we analyzed the chain length distribution of amylopectin by capillary electrophoresis (Fig. 5). The chain length distribution patterns of Taichung 65, Kinmaze, and *be1* were similar to each other, with a peak at DP 11 (Fig. 5a). On the other hand, *be2b* and *be1 be2b* showed a peak at DP 14 and also a broader peak at approximately DP 44, although the peak at DP 14 was lower and that at DP 44 was higher in *be1 be2b* that those in *be2b* (Fig. 5a). To compare the detailed differences in chain length distribution patterns, the patterns of WT cultivars were subtracted from those of mutant lines (Fig. 5b). The subtraction curve of *be1* (minus Taichung 65) showed an increase in DP 6–10 and DP 24–36, and a minor decrease in DP 37–54 (Fig. 5b), consistent with a previous study (Satoh et al. 2003b). The subtraction curve of *be2b* (minus Kinmaze) showed a considerable decrease in DP 6–13 and an increase in DP > 14 with broad peaks at DP 19 and DP 45 (Fig. 5b), consistent with previous studies (Nishi et al. 2001; Abe et al. 2014; Asai et al. 2014). The subtraction curve of *be1 be2b* (minus Kinmaze) also showed a great decrease in DP 6–16 and an increase in DP > 17, with broad peaks at DP 21 and DP 47 (Fig. 5b). When the subtraction curves of *be1 be2b* and *be2b* (minus Kinmaze) were compared, that of *be1 be2b* at DP > 10 shifted toward the longer side. Notably, *be1 be2b* showed less chains with DP 10–20 but more chains with DP > 21 than *be2b* (Fig. 5b).

To determine the apparent amylose content, true amylose content, and the extra-long chain content, and the ratio of long- to short-chains of amylopectin, we analyzed the debranched purified starch and isolated amylopectin by gel filtration chromatography (Fig. S1); the percentages are summarized in Table 3. The apparent amylose content (fraction I of starch) includes true amylose content and extra-long chains of amylopectin. Fraction II contains long-chains, and fraction III contains short-chains of amylopectin (Fig. S1) (Horibata et al. 2004). The apparent amylose contents of Taichung 65, Kinmaze, and *be1* were approximately 20%, while that of *be2b* was significantly higher than that of the WT by 26.5%, consistent with previous studies (Abe et al. 2014; Asai et al. 2014). Strikingly, the apparent amylose content of *be1 be2b* was 51.7%, which was almost double that of *be2b*. The content of extra-long chains of amylopectin in *be1 be2b* (1.3%) was less than in other lines including Taichung 65 (1.8%), Kinmaze (2.5%), *be1* (2.0%), and *be2b* (2.0%) (Table 3). Therefore, the true amylose content of *be1 be2b* was extremely high (50.5%). The ratio of short- to long-chain of amylopectin (fraction III/II) was approximately 3.0 (starch or amylopectin) in Taichung 65, Kinmaze, and *be1*; however, this value was 1.0 (starch) or 0.9 (amylopectin) in *be2b* and 0.5 (starch) or 0.6 (amylopectin) in *be1 be2b*, indicating that the content of long-chains of amylopectin was the highest in *be1 be2b*, followed by *be2b*.

**Table 3:** Apparent and true amylose contents and short/long amylopectin branch ratios of starch and amylopectin

Genotype	Fraction I (%)^*a*^		III/II ratio^*b*^		TAC (%)^*c*^
	Starch (AAC)	Amylopectin (ELC)	Starch	Amylopectin	Starch
WT (Taichung 65)	21.1 ± 0.3f	1.8 ± 0.1de	3.0 ± 0.0d	3.1 ± 0.1d	19.2
WT (Kinmaze)	21.6 ± 2.0f	2.5 ± 0.2d	2.8 ± 0.2d	3.2 ± 0.1d	19.1
*be1* (EM557)	21.6 ± 0.3f	2.0 ± 0.1d	3.2 ± 0.0d	3.3 ± 0.1d	19.5
*be2b* (EM10)	26.5 ± 0.7e	2.0 ± 0.1d	1.0 ± 0.0e	0.9 ± 0.1e	24.5
*be1 be2b* (#1403)	51.7 ± 0.4d	1.3 ± 0.1e	0.5 ± 0.0f	0.6 ± 0.0e	50.5

^a^Fraction I of starch analyzed by gel filtration chromatography represents the apparent amylose content (AAC), and that of purified amylopectin represents the extra-long chain content (ELC)

^b^III/II ratio represents the ratio of amylopectin short chains (fraction III) to amylopectin long chains (Fraction II). Data represent mean ± SE (*n* = 3). Each fraction was split as shown in Fig. S1

^c^True amylose content (TAC) was calculated by subtracting ELC from AAC

Different lowercase letters (d–f) indicate significant differences among rice genotypes (*P* < 0.05; Tukey-Kramer method)

### RS Content and Structure

Since *be1 be2b* accumulated high levels of amylose and long-chains of amylopectin, further experiments were conducted to analyze the content and structure of RS in various rice genotypes. RS contents were measured using raw and gelatinized rice flour as well as mashed and un-mashed cooked rice (Table 4). The content of RS was low in raw flour prepared from WT cultivars (Taichung 65 and Kinmaze) and *be1* single mutant (0.2% and 0.1%, respectively) but considerably higher in raw flour prepared from *be2b* and *be1 be2b* (11.6% and 35.1%, respectively). The RS contents of gelatinized rice flour prepared from WT cultivars and *be1* were 0.9% and 0.5%, respectively, which were slightly higher than the values obtained from the raw rice flour of these genotypes. In *be2b*, the RS content of gelatinized rice flour was one-third (3.9%) of that of raw rice flour, whereas in *be1 be2b*, the RS content of gelatinized rice flour was three quarters (26.6%) of that of raw rice flour. The content of RS in un-mashed cooked rice prepared from Taichung 65, Kinmaze, and *be1* was low (1.3%, 1.2%, and 1.4%, respectively) but considerably higher in un-mashed cooked rice prepared from *be2b* and *be1 be2b* (27.5% and 76.2%, respectively). The RS content of *be2b* (EM10) in un-mashed cooked rice in the previous report (Itoh et al. 2017) was 21.6%, which was lower than this study (Table 4). This difference may be caused by the difference in amount of water added to steam rice (water was 1.8 and 1.5 folds of the weight of polished rice in previous report and current study, respectively). The RS contents of mashed cooked rice prepared from WT cultivars (Taichung 65 and Kinmaze) and *be1* were 0.9% and 1.2%, respectively, which were slightly lower than those of un-mashed cooked rice. By contrast, the RS contents of mashed cooked rice prepared from *be2b* and *be1 be2b* were 10.8% and 28.4%, respectively, which were reduced to approximately 40% compared with the RS contents of un-mashed cooked rice.

**Table 4:** RS contents of raw and gelatinized rice flour and mashed and un-mashed cooked rice

Genotype	Raw rice flour	Gelatinized rice flour	Un-mashed cooked rice	Mashed cooked rice
WT (Taichung 65)	0.2 ± 0.0c	0.9 ± 0.0c	1.3 ± 0.1c	0.9 ± 0.0c
WT (Kinmaze)	0.2 ± 0.0c	0.9 ± 0.0c	1.2 ± 0.0c	0.9 ± 0.0c
*be1* (EM557)	0.1 ± 0.0c	0.5 ± 0.0c	1.4 ± 0.3c	1.2 ± 0.0c
*be2b* (EM10)	11.6 ± 0.2b	3.9 ± 0.0b	27.5 ± 1.6b	10.8 ± 0.2b
*be1 be2b* (#1403)	35.1 ± 1.0a	26.6 ± 0.2a	76.2 ± 1.4a	28.4 ± 0.4a

Data represent mean ± SE (*n* = 3). Different lowercase letters (a–c) indicate significant differences among rice genotypes (*P* < 0.05; Tukey-Kramer method)

**Fig. 6:**
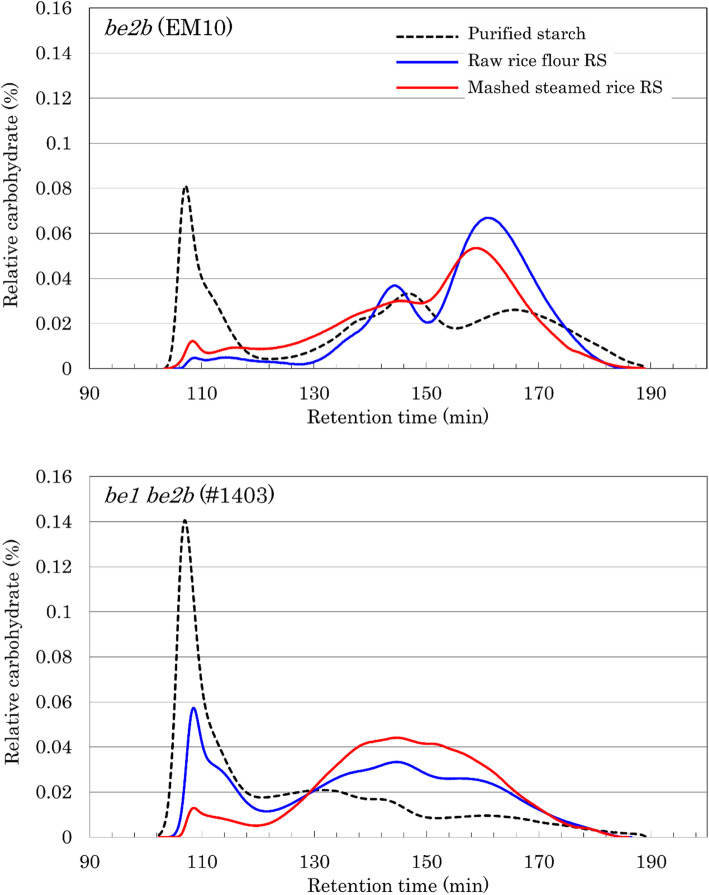
Structure of resistant starch (RS) in raw rice flour and mashed cooked rice. RS samples prepared from *be2b* and *be1* *be2b* were debranched by isoamylase treatment and analyzed by gel filtration chromatography. The difference in retention time (RT) to Fig. S1 was due to different lot of the column

To determine RS structure, RS was purified from raw rice flour and mashed cooked rice of high RS lines, *be2b* and *be1 be2b*. The RS samples were solubilized in DMSO, debranched, analyzed by gel filtration chromatography, and compared with the elution profile of purified starch (Fig. 6). In *be2b*, the elution profile of purified starch showed peak amylose, amylopectin long chains, and amylopectin short chains at 107, 146, and 166 min, respectively (Fig. 6). Although the same three peaks were observed in the elution profile of RS obtained from raw rice flour of *be2b*, the first peak of RS was negligible compared with that of purified starch; by contrast, the second and third peaks of RS (obtained at 144 and 160 min, respectively) were higher than those of purified starch, with a shift toward higher molecular weight. In the elution profile of RS obtained from mashed cooked rice of *be2b*, the first peak was slightly higher than that of RS from raw rice flour but much lower than that of purified starch. The elution profile of RS obtained from mashed cooked rice of *be2b* did not show a clear second peak but rather a broad elution pattern. The third peak of RS obtained from mashed cooked rice of *be2b* was higher than that of purified starch but lower than that of RS from raw rice flour, and was obtained at a higher molecular weight at 158 min. In contrast to *be2b*, the elution pattern of *be1 be2b* purified starch showed a very high amylose peak at 107 min, broad peak at 120–140 min, low peak of amylopectin long chains at 144 min, and very low peak of amylopectin short chains at 160 min. The elution profile of RS obtained from *be1 be2b* raw rice flour showed three peaks; the area of the first peak was 30–40% of the area of purified starch, while the second and third peaks were higher than those of purified starch. The elution profile of RS obtained from *be1 be2b* mashed cooked rice showed only two peaks; the first peak was considerably reduced compared with that of purified starch, while the second peak (at 146 min) was high and appeared to be amalgamated.

## Discussion

### Generation of Non-transgenic Rice Lines Deficient in BEI and BEIIb

Rice is one of the top three crops with the highest production in the world, and serves as the major dietary source of carbohydrates in Asian and African countries (Fairhurst and Dobermann 2002; Awika 2011). Given the recent increase in the number of diabetic patients worldwide, new rice lines allowing the restriction of carbohydrate intake are being developed (Wang et al. 2017). Among the various starch biosynthetic enzymes, it is clear that reduction in the level of amylopectin biosynthesis enzymes, BEIIb and/or SSIIIa, leads to high amylose content (Fujita 2014; Wang et al. 2017). Importantly, suppression of multiple BE isozymes allows the accumulation of amylose and RS to high levels in maize (Li et al. 2008), wheat (Regina et al. 2006; Corrado et al. 2020), barley (Regina et al. 2010; Carciofi et al. 2012), and rice (described later). In a rat feeding trial, intake of RS improved gastrointestinal health (Regina et al. 2006; Zhu et al. 2012), and prevented sudden increase in blood glucose levels (Zhu et al. 2012). In rice, the expression of *BEI* and *BEIIb* genes has been suppressed using anti-sense RNA (Wei et al. 2010), artificial microRNA (Butardo et al. 2011), and RNA interference (RNAi) technology (Sawada et al. 2018), and the resulting transgenic lines showed drastic starch phenotypes compared with the WT. However, there are no information about the seed weight or agricultural traits in these transgenic rice lines, except that the materials produced by RNAi technology, which suppressing BEI and BEIIb, significantly reduced it seed weight (Sawada et al. 2018). Unlike these previous studies, we generated a non-transgenic *be1 be2b* double mutant rice line for more practical use by crossing single mutant lines that are completely deficient in BEI or BEIIb. Therefore, the effects of loss of these enzymes are more prominent compared with the previously generated transgenic rice lines which have low levels of these enzymes, although it cannot be compared directly due to the difference of the methods.

The *be1 be2b* double mutant showed higher seed weight than the *be2b* single mutant (Fig. 2) over three consecutive years (2016, 2017, and 2018; data not shown). There are at least three reasons why the seed weight of *be1 be2b* was heavier than that of *be2b* single mutant. The first possibility is that the absence of both BEI and BEIIb diminished amylopectin biosynthesis but enhanced amylose synthesis. Loss of BEIIb is accompanied by the reduction in SSI activity to 50%, which may be due to the absence of branches that serve as primers for SSI. SSI activity in *be1 be2b* was also lower than that in the WT (Miura et al., in preparation). Less amylopectin branches in *be1 be2b* result in less non-reducing ends to serve as a substrate for SS. This leads to less consumption of ADP-glucose by SS isozymes including SSI. Then, an abundance of ADP-glucose in amyloplasts enhances amylose biosynthesis by granule-bound starch synthase I (GBSSI). The second possibility is that *be1 be2b* may have inherited some seed weight traits from Taichung 65. The seed weight of Taichung 65, the parental WT genotype of *be1*, was slightly (1.1-fold) heavier than that of Kinmaze, the parental WT genotype of *be2b* (Fig. 2), and this tendency was observed in all 3 years (2016, 2017, and 2018; data not shown). The third possibility is the difference in flowering time. Plants of *be2b* (Kinmaze background) flowered in late August to early September, whereas those of *be1* (Taichung 65 background) and *be1 be2b* flowered in mid-August in Akita, Japan. Therefore, it is possible that the *be1 be2b* double mutant inherited flowering loci from *be1* (Taichung 65 background). Earlier flowering time guarantees higher temperature during the seed development, which results in higher activity of starch biosynthetic enzymes, thus enhancing starch synthesis.

In addition, the growth (Fig. 3) and fertility rate of *be1 be2b* plants (Table 1) was similar to those of the WT. These data suggest that the presence of BEIIa alone is sufficient for plant growth and the accumulation of up to 60% endosperm starch compared with the WT. The agricultural traits of *be1 be2b* such as seed weight and growth are beneficial for commercializing the double mutant as an ultra-high RS rice cultivar, although further breeding such as a backcrossing with elite rice cultivars is necessary. Back-crossing of *be1 be2b* mutant to the high yield elite rice cultivar is currently being conducted and its offspring is selected using molecular makers generated against the mutation sites. It is hoped that the back-crossed lines will be used widely as practical high RS rice cultivars in near future.

### Effects of Loss of both BEI and BEIIb on Amylopectin Structure and Thermal Properties

It is clear that the loss of BEIIb, among the three BE isozymes, drastically affects starch properties by decreasing amylopectin short chains (Nishi et al. 2001; Nakamura 2002; Takahashi and Fujita 2017). The *be1 be2b* double mutant synthesized less amylopectin chains with DP 10–20 and more amylopectin chains with DP > 20 than the *be2b* single mutant (Fig. 5a and b), resulting in a higher ratio of long to short amylopectin branches (Table 3). These results are consistent with the GEMS-0067 lines in maize, which are thought to be *be1 be2b* double mutants (Li et al. 2008), and with the down-regulation of *BEI* and *BEIIb* genes in transgenic rice (Zhu et al. 2012; Sawada et al. 2018; Lin et al. 2019). Comparison of the theoretical and actual subtraction curves of chain length distribution showed that the theoretical curve obtained by the addition of the subtraction curves of *be1* and *be2b* single mutants [(*be1* – WT) + (*be2b* – WT)] were less chains with DP 10–20 and more chains with DP ≥ 40 (Fig. 5b) than that of the actual curve of *be1 be2b* (*be1 be2b* – WT). This indicates a synergistic effect due to the loss of both BE isozymes, suggesting that in the absence of BEIIb, the remaining BE enzymes (BEI and/or BEIIa) may generate amylopectin branches with DP 10–20; however, when both BEI and BEIIb are absent, BEIIa alone may not be able to compensate for their absence, and the number of branches with DP 10–20 may also be diminished. The average and median values of amylopectin chain length distribution (Table S1) were DP 26–27 and DP 14, respectively, in WT cultivars, and DP 33.8 and DP 17 in *be2b*; however, both values were greater in *be1 be2b* (average, DP 36.7; median, DP 19), suggesting that amylopectin possessed extremely long double-helical branch chains in the absence of BEI and BEIIb.

These unique starch structures greatly influence the gelatinization temperature. High temperature and energy are required to unwind double-helical structure (Hizukuri 1986); therefore, the longer the amylopectin branches, the higher the gelatinization temperature. Most of double helices in amylopectin molecules consist of amylopectin branches with DP ≤ 24 within a cluster of amylopectin. The greater the number of short amylopectin chains (DP ≤ 12) and the lower the number of intermediate chains (13 ≤ DP ≤ 24), the lower the gelatinization temperature (Fujita 2014; Hayashi et al. 2015). The increase in the gelatinization temperature of *be2b* by 16 °C compared with the WT may be because *be2b* showed a significant decrease in amylopectin with DP ≤ 12 and a great increase in amylopectin with DP 13–24 (Table S1). Although *be1 be2b* had slightly less branches with DP ≤ 12 compared with *be2b*, branches with DP 13–24 in *be1 be2b* were significantly less than those in *be2b* (Table S1). However, the peak gelatinization temperature of *be1 be2b* was significantly higher than that of *be2b* by 7.5 °C. The amylopectin chain length responsible for gelatinization temperature is essentially DP ≤ 24 in the WT as described above. However, formation of even longer double helices in *be2b* (EM10) and *be1 be2b* likely resulted in an extremely high gelatinization temperature due to the significant increase in DP 25–36 in *be1 be2b* compared with that of *be2b* (Table S1). These trends are in agreement with the results of previous studies on maize GEMS-0067 lines (Li et al. 2008) and transgenic rice lines down-regulated for the expression of *BEI* and *BEIIb* genes (Lin et al. 2019).

### Effects of Loss of both BEI and BEIIb on Amylose Content

The apparent amylose content of *be1 be2b* was 51.7%, as measured by gel filtration chromatography. This value was higher than the current highest record of 45.1% in the non-transgenic *ss3a be2b* double mutant *japonica* rice (#4019) (Asai et al. 2014). The amylose content of a transgenic *indica* rice line, in which the expression of *BEI* and *BEIIb* was down-regulated, was 44.8%, based on gel filtration chromatography (Zhu et al. 2012), while that of maize GEMS-0067 lines was 83.1–85.6%, based on the iodine method (Li et al. 2008). Suppression of both *BEI* and *BEIIb* genes leads to higher amylose content compared with the suppression of *BEIIb* alone, which is in agreement with previous studies (Li et al. 2008; Wang et al. 2018). In these cases, loss of two major branching enzymes, BEI and BEIIb, involved in amylopectin biosynthesis likely resulted in enhanced amylose biosynthesis.

Rice cultivars with high amylose content can be divided into two types: high true amylose content cultivars and high extra-long amylopectin chain content cultivars (Horibata et al. 2004). Extra-long chain is a long linear glucan, similar to amylose but connected to amylopectin, synthesized by GBSSI which is encoded by *Waxy* (*Wx*) gene (Takeda et al. 1987). The difference in extra-long chain content is thought to be dependent on single nucleotide polymorphisms (SNPs) in the *Wx g*ene (Crofts et al. 2019). The *be1 be2b* double mutant used in this study was low in extra-long chain (1.3%) (Table 3, Fig. S1) but high in true amylose content (50.5%). This was because *be1 be2b* harbors the *Wx*^*b*^ (*gbss1*^*L*^) gene; this gene was inherited from either Kinmaze or Taichung 65 and contains a thymine instead of a guanine at the first nucleotide position of intron 1, resulting in a low level of GBSSI protein (Crofts et al. 2019).

The maize GEMS-0067 lines accumulate higher levels of amylose and intermediate components than the *amylose-extender* (*ae*) lines, in which intermediate components, i.e., glucan molecules, are smaller than normal amylopectin but are enriched with longer average branch chains (Li et al. 2008). The elution profile of debranched purified starch obtained by gel filtration chromatography showed that amylose in *be1 be2b* was eluted as a broad peak at 110–120 min, which did not return completely to the baseline, unlike that observed in *be2b* (Fig. S1). On the other hand, the elution profile of purified amylopectin in *be1 be2b* showed almost no peak around 110 min, indicating that glucans eluted from the purified starch at approximately 110–120 min may be low molecular weight amylose. Thus, starch in the rice *be1 be2b* double mutant may have some different characteristics from the intermediate chains in maize GEMS-0067 lines. The apparent amylose content (83–86%) and extra-long chain content (20–26.8%) of maize GEMS-0067 lines were much higher than those of rice *be1 be2b*, as measured by the gel filtration method (Li et al. 2008). Furthermore, suppression of all three genes encoding BE isozymes (*BEI*, *BEIIa*, and *BEIIb*) in transgenic barley resulted in a further increase in amylose content (99.1%), as measured by the iodine method (Carciofi et al. 2012). Considering these cases, amylose content of *be1 be2b* was relatively low; however, this is because *be1 be2b* harbors the *Wx*^*b*^ gene that encodes low levels of GBSSI (Sano 1984), whereas maize and barley lines harbor the normal *Wx* gene, which encodes high levels of GBSSI. On the other hand, when both BEI and BEIIb activities were suppressed in transgenic *indica* rice harboring the *Wx*^*a*^ gene, which encodes high levels of GBSSI, the amylose content was 64.8% as determined by the iodine method (Zhu et al. 2012). Although the amylose content measured by different methods cannot be directly compared, regardless of the level of GBSSI, rice *be1 be2b* including previous and our studies seems to contain lower amylose content than other plant species. This suggests that additional factors prevent excess biosynthesis or accumulation of amylose in rice.

### Effects of Loss of both BEI and BEIIb on RS Content

Amylopectin, the major component of starch, forms double helices using adjacent branches in its native state. When starch absorbs water and gets heated, it undergoes gelatinization. The double helices are then unwound, and gelatinized starch is easily broken down by digestive enzymes. When the gelatinized starch is cooled, nearby branches re-form double helices to produce retrograded starch, although those double helices may be imperfect. When the amylopectin branch chains are long, starch is harder to gelatinize, quicker to retrograde, and therefore less degradable. By contrast, when the amylopectin branch chains are short, starch is easier to gelatinize, slower to retrograde, and therefore more degradable. Previously, we showed that the loss of BEIIb increases the amount of long amylopectin chains, which form long double helices, leading to a significant increase in RS content compared with high amylose rice lines such as indica rice and *SSIIIa* deficient mutant rice (Tsuiki et al. 2016; Zhou et al. 2016).

In this study, the *be2b* single mutant showed significantly higher RS content than WT cultivars and *be1*, regardless of the product type and the method of preparation (raw or gelatinized rice flour and mashed or un-mashed cooked rice). Strikingly, the *be1 be2b* double mutant showed 2.6–6.8-fold higher RS content than *be2b* (Table 4), possibly because of the high apparent amylose content as well as high amylopectin long branches in *be1 be2b*. This means that the amounts of *be1 be2b* required to produce a food product with a certain level of RS content is only one-third to seventh of the *be2b*. These findings in non-transgenic *japonica* rice, *be1 be2b*, are consistent with those in maize GEMS-0067 lines (Jiang et al. 2010) as well as in transgenic *japonica* rice lines suppressed from the expression of *BEI* and *BEIIb* genes (Lin et al. 2019).

Rice is utilized in many different food applications, in addition to the ordinary cooked rice. To reflect those differences in applications, the RS contents were analyzed using samples prepared by four different methods. Although chewing of foods greatly varies among individuals, the RS content of cooked rice would be intermediate between that of un-mashed and completely mashed cooked rice. Additionally, although the degree of gelatinization may vary with the application, such as bread, cookies, and noodles, the RS content of rice flour would be intermediate between that of raw and completely gelatinized rice flour. Comparison of RS content of rice samples prepared by different methods revealed that the RS content of all analyzed rice lines was the highest in un-mashed cooked rice (Table 4). This was perhaps because the digestive enzymes could not function effectively in un-mashed cooked rice, given the small surface area, and this is thought to be a typical feature of RS1. In the WT and *be1* mutant, the second highest RS content was detected in mashed cooked rice and gelatinized rice flour, while the lowest RS content was detected in raw rice flour (Table 4), possibly because the digestive enzymes functioned effectively in raw flour, given its large surface area. It has been suggested that the majority of starch in the raw rice flour can be digested by α-amylase, even though it has A-type crystallinity. The reason why gelatinized rice flour showed higher RS content than raw rice flour may be because grinding with pestle and mortar after gelatinization resulted in the samples becoming sticky and slightly lumpy, leading to lower surface area for digestive enzymes. On the other hand, in *be2b* and *be1 be2b*, the second highest RS content was detected in raw rice flour, followed by mashed cooked rice, and the least in gelatinized starch. The reason why raw rice flour showed higher RS content than mashed cooked rice and gelatinized rice flour may be explained by two possibilities. The loss of BEIIb in *be2b* and *be1 be2b* mutants probably increased long amylopectin chains, which formed longer double helices with B-type crystallinity and were more resistant to degradation by digestive enzymes. The other possibility is that gelatinized rice flour and mashed cooked rice prepared from *be2b* and *be1 be2b* were rapidly retrograded; this is thought to be a typical feature of RS3. Although some lumps appeared during the grinding procedure, the lumps of *be2b* and *be1 be2b* were less sticky than that of the WT and *be1* and could be easily suspended in the digestive enzyme solution; therefore, samples had a larger surface area for digestive enzymes to work effectively.

### Effects of Loss of both BEI and BEIIb on the Structure of RS

Detailed analyses of RS structure have been performed only in maize GEMS-0067 and *ae* lines (Jiang et al. 2010). RS samples of GEMS-0067 lines treated with thermostable α-amylase at 95–100 °C contained two components: high molecular weight glucans (DP 840–951), including amylose and slightly branched glucans derived from intermediate components, and linear, low molecular weight glucans (DP 59–74) (Jiang et al. 2010). In this study, RS samples were prepared by treating rice samples with digestive enzymes at 37 °C. The remaining RS materials were debranched and analyzed by gel filtration chromatography (Fig. 6). The RS structure of *be2b* rice flour showed almost no amylose peak, while that of *be1 be2b* rice flour showed a clear amylose peak, although the peak area of *be1 be2b* RS from raw rice flour was 30–40% of the peak area of *be1 be2b* purified starch. This may be because *be1 be2b* has a very high true amylose content (50.5%), and the amylose may have formed long double helices. Of the true amylose content, 30–40% could not be degraded by digestive enzymes and therefore maintained the original molecular weight of amylose, while 60% was partially degraded to lower molecular weight amylose; hence, the peaks at 145 and 160 min, corresponding to amylopectin long chains and amylopectin short chains, respectively, were higher than those of purified starch. It is also possible that the partially degraded amylose, which was of a similar molecular weight as the long amylopectin chains, formed double helices, thus avoiding degradation by digestive enzymes. The RS sample of *be1 be2b* rice flour showed a small peak at 160 min. This peak is thought to represent the remaining long amylopectin chains within the size of one amylopectin cluster, and corresponds to RS2. The first peak of the RS structure of mashed cooked rice from *be1 be2b* was diminished similar to that of *be2b*. This suggests that almost all gelatinized amylose can be degraded to lower molecular weight amylose. The peak at 120–130 min corresponding to low molecular weight amylose from purified starch was lower in RS prepared from mashed cooked rice of *be1 be2b* than those of purified starch. It can be speculated that the low molecular weight amylose can also be degraded to an even lower molecular weight. The RS prepared from mashed cooked rice of *be2b* showed a clear peak at 158 min, which corresponds to long amylopectin chains within the size of one amylopectin cluster, as described above. By contrast, the RS prepared from mashed cooked rice of *be1 be2b* showed a large peak at 130–170 min, with the tallest peak at 144 min. We speculate that this peak represents the degradation products of amylose, long amylopectin chains spanning two or more clusters, and chains within one cluster. The gelatinized starch was incubated with digestive enzymes at 37 °C. It is possible that starch retrograded and formed double helices with a variety of different sized glucans, thus transforming into RS3, which is more resistant to degradation by digestive enzymes. The type of glucan molecules that form double helices in raw rice flour and retrograded starch can only be speculated at this moment, and further detailed analyses of RS structure are required. In addition, the actual RS content and structure in processed foods should also be determined in future studies.

## Conclusion

The *be1 be2b* rice mutant completely lacking both enzymes at the protein levels was generated. Seed weight of *be1 be2b* mutant was approximately 60% of the wild type and rather heavier than that of *be2b* mutant over the past three consecutive years. The *be1 be2b* mutant showed a decrease in intermediate amylopectin chains with DP10–20 and an increase in long amylopectin chains with DP > 21 compared with *be2b*. The amylose content of *be1 be2b* mutant (51.7%) was the highest among all pre-existing non-transgenic rice lines. The RS contents of mashed cooked rice and raw rice flour of *be1 be2b* mutant were 3-fold higher than those of *be2b* mutant. Gel-filtration analyses of starch treated with digestive enzymes showed that the RS in *be1 be2b* mutant was composed of the degradation products of amylose and long amylopectin chains. *be1 be2b* generated in this study must serve as a good material for an ultra-high RS rice cultivar that expected of lifestyle-related diseases such as diabetes all over the world.

## Methods

### Plant Materials

Rice (*Oryza sativa* L.) *be1* and *be2b* single mutant lines, EM557(*be1*) (Satoh et al. 2003b) and EM10 (*be2b*) (Nishi et al. 2001), respectively, were previously isolated from N-methyl-N-nitrosourea (NMU)-mutagenized populations of *japonica* WT cultivars of Taichung 65 and Kinmaze, respectively. *be1* (EM557) possesses a single nucleotide mutation at the end of exon 10 of *BEI* gene, which introduces a stop codon into exon 11, resulting in no BEI protein (unpublished data). *be2b* possesses a single nucleotide mutation at the end of intron 9 of *BEIIb* gene, which produces incorrect mRNA (EM10, Wada et al., in preparation). The *be1* and *be2b* single mutants were crossed, and the F_2_ population was screened. Opaque seeds were analyzed by western blotting (Fig. 1), and a double *be1 be2b* mutant line, namely #1403, were selected. F_4_ and F_5_ seeds were used for subsequent analysis. All rice lines were grown in a paddy field of Akita Prefectural University during the summer months under natural conditions.

### Western Blotting

Single mature rice grains without embryo were ground to a fine powder. Total proteins were extracted using 300 μL sample buffer containing 125 mM Tris-HCl (pH 6.8), 8 M urea, 4% (w/v) sodium dodecyl sulfate (SDS), 5% (v/v) β-mercaptoethanol, and 0.05% (w/v) bromophenol blue. Samples were continuously agitated for 3 h, and then centrifuged at 15,000 rpm (20,000 *g*) at room temperature for 10 mins. The supernatant of each sample was loaded onto a 7.5% polyacrylamide gel and separated by SDS-polyacrylamide gel electrophoresis (PAGE). Proteins in the gel were blotted onto a polyvinylidene fluoride (PVDF) membrane and detected by anti-BEI (Satoh et al. 2003b) and anti-BEIIb (Nishi et al. 2001) antisera with 1:2000 and 1:5000 dilutions, respectively, as described previously (Crofts et al. 2012; Crofts et al. 2015).

### Seed Morphology

The morphology of brown rice grains was observed as described previously (Miura et al. 2018). using a stereomicroscope (SZX7-ILST-0 (SP), OLYMPUS, Tokyo, Japan), with a light source from above and beneath. Photos were taken using a digital camera.

### Starch Analyses

Purified starch was extracted from polished rice grains using the cold-alkaline method, as described previously (Yamamoto et al. 1973; Yamamoto et al. 1981). Purified starch granules were coated with gold using a fine coater (JEOL JFC-1200, Tokyo, Japan) for 120 s. Starch granule morphology was observed by scanning electron microscopy (SEM; HITACHI TM3030Plus, Tokyo, Japan) in a secondary electron mode at 15 kV, according to the method of Fujita et al. (2003). To perform X-ray diffraction measurements, purified starch isolated using the above method was equilibrated in a chamber at room temperature and 100% relative humidity for 24 h. The X-ray diffraction patterns of starch were measured using copper, nickel foil-filtered, Kα radiation with the X-ray diffractometer (MiniFlex600 Rigaku, Tokyo, Japan) at 40 kV and 20 mA. The scanning region of two-theta angle (*2θ*) varied from 5.0° to 40.0°, with a scanning speed of 0.3° s^− 1^ (Fujita et al. 2006). The relative starch crystallinity (RSC) of each endosperm starch from the mutant lines was determined by the method described in a previous report (Kodama et al. 2011; Abe et al. 2013) using calcium fluoride as an internal standard. The thermal properties of purified starch were determined by differential scanning calorimetry (DSC; Seiko Instrument 6100, Chiba, Japan) and analyzed as described previously (Fujita et al. 2003; Fujita et al. 2006).

### Starch Structure Analyses

The chain length distribution of endosperm starch was determined by capillary electrophoresis (P/ACE MDQ Plus Carbohydrate System, AB Sciex, Framingham, Ma, USA), according to the method of Fujita et al. (2001). Amylopectin was isolated from 1 g purified starch using n-butanol and isoamyl alcohol, as described previously (Schoch 1957; Takeda et al. 1986). The purified starch and isolated amylopectin were debranched using *Pseudomonas* isoamylase (Hayashibara, Okayama, Japan) and analyzed by gel filtration chromatography (Toyopearl HW-55S and HW-50S × 3, Tosoh, Tokyo, Japan) (Horibata et al. 2004; Fujita et al. 2007; Toyosawa et al. 2016). The apparent amylose content, ratio of short- to long-chain amylopectin, true amylose content, and extra-long-chain amylopectin content were calculated as described previously (Horibata et al. 2004; Fujita et al. 2007; Toyosawa et al. 2016).

### Measurement of RS Content

RS contents were analyzed in cooked rice (mashed and un-mashed) and rice flour (raw and gelatinized). To prepare cooked rice, 1 g polished rice was washed twice with distilled water and cooked with 1.5 volumes of water (w/w of dry polished rice) in 15 mL plastic test tubes using a rice cooker (NS-WF10, Zojirushi, Osaka, Japan). Then, 250 mg mashed and un-mashed cooked rice were transferred to new tubes. To prepare rice flour, polished rice was ground to a fine powder using a mortar and pestle, and sieved through a 100 μm colander. For the raw rice flour test, 100 mg rice flour was transferred to a tube. For the gelatinized rice flour test, 100 mg rice flour was boiled with two volumes of water (w/w) for 10 mins and the resulting rice pastes were ground again with a pestle and mortar. Samples were digested in 4 mL digestive enzymes using the RS assay kit (Megazyme, Bray, Ireland) containing porcine pancreatic α-amylase (120 U) and amyloglucosidase (12 U) at 37 °C for 16 h by continuous shaking. RS contents were measured as described previously (Tsuiki et al. 2016).

### Analysis of RS Structure

Mashed cooked rice and raw rice flour (1 g each) were digested in 40 mL digestive enzyme solution, as described above, and precipitated with an equal volume of ethanol. Samples were then centrifuged at 3000 rpm (937 *g*) at room temperature for 10 mins. The precipitate containing RS was washed with 40 mL of 50% ethanol. The precipitation and centrifugation steps were repeated once again. The RS pellet was dissolved in 100% dimethyl sulfoxide (DMSO) and boiled for 1 h, while shaking every 10 mins. The RS solution was centrifuged at 3000 rpm (937 *g*) at room temperature for 10 mins. The RS samples were precipitated by mixing with three volumes of 100% ethanol and incubated at − 30 °C for 1 h. The RS materials were centrifuged at 3000 rpm (937 *g*) at 4 °C for 10 mins, and the resulting pellet was suspended in 5 mL of 100% ethanol. After centrifugation at 3000 rpm (937 *g*) at 4 °C for 10 mins, the RS pellets were dried in a speed vacuum. The dried samples were ground to a fine powder, and 3–5 mg of the powdered RS material was gelatinized in 1 N NaOH and neutralized with 1 N HCl. Then, the gelatinized RS was debranched using *Pseudomonas* isoamylase (Hayashibara, Okayama, Japan) to perform gel filtration analysis, as described above.

### Data Analyses

DSC, carbohydrate contents of each fraction by gel-filtration chromatography, RS contents, RSC by X-ray diffraction and percentages and average of chain length of amylopectin by capillary electrophoresis data were statistically analyzed by a one-way variance (ANOVA) and Tukey-Kramer method (*p* < 0.05). Significant difference of seed weight between *be2b* and *be1 be2b* was tested by Student’s t-test (*p* < 0.05).

## Supplementary Information

**Additional file 1: Table S1.** Percentages and average chain length of amylopectin branches analyzed by capillary electrophoresis.

**Additional file 2: Fig. S1.** Elution profiles of debranched starch and amylopectin analyzed by gel filtration chromatography. Fraction I contains amylose or extra-long amylopectin chains. Fraction II contains long amylopectin chains. Fraction III contains short amylopectin chains. Red lines indicate patterns obtained from starch, and blue lines indicate patterns obtained from the purified amylopectin. Differences in retention time (RT) in Kinmaze were due to difference of the lot of the column.

## Data Availability

Not applicable.
