# Supplementary material for: Generation and Starch Characterization of Non-Transgenic BEI and BEIIb Double Mutant Rice (Oryza sativa) with Ultra-High Level of Resistant Starch
Source: Rice (N Y). 2021 Jan 6;14:3. doi: 10.1186/s12284-020-00441-0 (PMC7788159; doi:10.1186/s12284-020-00441-0)
Supplement: Supplementary file 1 — Additional file 1: Table S1. Percentages and average chain length of amylopectin branches analyzed by capillary electrophoresis. [file 12284_2020_441_MOESM1_ESM.docx]

**Table S1.** Percentages and average chain length of amylopectin branches analyzed by capillary electrophoresis.

| Genotype | Percentage of amylopectin branches  (DP5-70) | | | | Average DP | Median DP |
| --- | --- | --- | --- | --- | --- | --- |
|  | DP ≤ 12 | DP 13–24 | DP 25–36 | DP ≥ 37 |  |  |
| WT (Taichung 65) | 37.0 ± 0.1a | 47.7 ± 0.1c | 8.0 ± 0.0d | 7.2 ± 0.0c | 27.0c | 14 |
| WT (Kinmaze) | 38.6 ± 0.3a | 47.2 ± 0.0c | 7.7 ± 0.2d | 6.5 ± 0.1c | 26.3c | 14 |
| *be1* (EM557) | 38.8 ± 0.1a | 45.8 ± 0.0d | 8.6 ± 0.0c | 6.8 ± 0.0c | 26.7c | 14 |
| *be2b* (EM10) | 20.0 ± 1.0b | 53.8 ± 0.4a | 11.7 ± 0.2b | 14.5 ± 0.5b | 33.8b | 17 |
| *be1 be2b* (#1403) | 17.3 ± 0.2b | 50.1 ± 0.3b | 14.8 ± 0.2a | 17.8 ± 0.3a | 36.7a | 19 |

Data represent mean ± standard error (*n* = 3). DP, degree of polymerization. Different lowercase letters (a–d) indicate significant differences among rice genotypes (*P* < 0.05; Tukey-Kramer method).
