# Supplementary material for: Generation and Starch Characterization of Non-Transgenic BEI and BEIIb Double Mutant Rice (Oryza sativa) with Ultra-High Level of Resistant Starch
Source: Rice (N Y). 2021 Jan 6;14:3. doi: 10.1186/s12284-020-00441-0 (PMC7788159; doi:10.1186/s12284-020-00441-0)

**Fig. S1.** Elution profiles of debranched starch and amylopectin analyzed by gel filtration chromatography. Fraction I contains amylose or extra-long amylopectin chains. Fraction II contains long amylopectin chains. Fraction III contains short amylopectin chains. Red lines indicate patterns obtained from starch, and blue lines indicate patterns obtained from the purified amylopectin. Differences in retention time (RT) in Kinmaze were due to difference of the lot of the column.


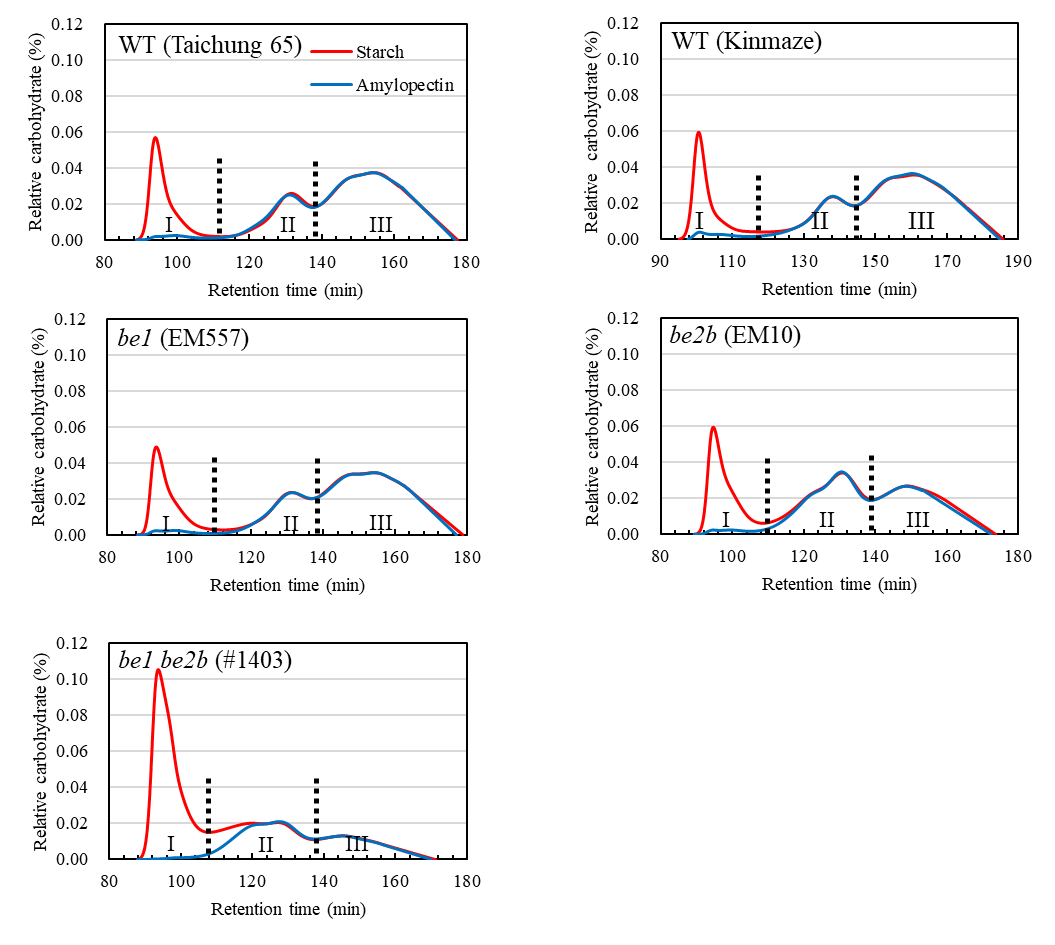

Supplement: Supplementary file 2 — Additional file 2: Fig. S1. Elution profiles of debranched starch and amylopectin analyzed by gel filtration chromatography. Fraction I contains amylose or extra-long amylopectin chains. Fraction II contains long amylopectin chains. Fraction III contains short amylopectin chains. Red lines indicate patterns obtained from starch, and blue lines indicate patterns obtained from the purified amylopectin. Differences in retention time (RT) in Kinmaze were due to difference of the lot of the column. [file 12284_2020_441_MOESM2_ESM.docx]
